# Methylation-mediated silencing of *EDN3* promotes cervical cancer proliferation, migration and invasion

**DOI:** 10.3389/fonc.2023.1010132

**Published:** 2023-02-03

**Authors:** Peng Zhu, Xiang Li, Yujie Liu, Jing Xiong, Ding Yuan, Yan Chen, Lili Luo, Ju Huang, Binbin Wang, Quanfang Nie, Shuli Wang, Liying Dang, Shu Li, Yan Shu, Wei Zhang, Honghao Zhou, Lan Fan, Qing Li

**Affiliations:** ^1^ Department of Clinical Pharmacology, Xiangya Hospital, Central South University, Changsha, China; ^2^ Institute of Clinical Pharmacology, Central South University, Hunan Key Laboratory of Pharmacogenetics, Changsha, China; ^3^ Engineering Research Center of Applied Technology of Pharmacogenomics, Ministry of Education, Changsha, China; ^4^ Department of Gynaecology, The Third Xiangya Hospital, Central South University, Changsha, China; ^5^ Department of Gynaecology and Obstetrics, The Second Xiangya Hospital, Central South University, Changsha, China; ^6^ Health Management Center, Xiangya Hospital, Central South University, Changsha, China; ^7^ Xiangya Medical Laboratory, Central South University, Changsha, China; ^8^ Department of Gynaecology, The First Affiliated Hospital of Shantou University Medical College, Shantou, China; ^9^ Department of Obstetrics and Gynecology, Loudi Central Hospital, Loudi, China; ^10^ Department of Obstetrics and Gynecology, Zhengzhou Central Hospital Affiliated to Zhengzhou University, Zhengzhou, China

**Keywords:** EDN3, DNA methylation, cervical cancer, CIN, biomarker

## Abstract

Cervical cancer (CC) remains one of the leading causes of cancer-related deaths worldwide. However, cervical cancer is preceded by the pre-malignant cervical intraepithelial neoplasia (CIN) that can last for up to 20 years before becoming malignant. Therefore, early screening is the key to prevent the progression of cervical lesions into invasive cervical cancer and decrease the incidence. The genes, down-regulated and hypermethylated in cancers, may provide potential drug targets for cervical cancer. In our current study, using the datasets from Gene Expression Omnibus (GEO) and the Cancer Genome Atlas (TCGA) databases, we found that endothelin 3 (*EDN3*) was downregulated and hypermethylated in cervical squamous cell carcinoma (CSCC). The further analysis in GSE63514 (n=128) dataset and in our samples (n=221) found that the expression of *EDN3* was decreased with the degree of cervical lesions. Pyrosequencing was performed to evaluate 4 CpG sites of the *EDN3* promoter region in our samples (n=469). The data indicated that the methylation level of *EDN3* was increased with the degree of cervical lesions. *EDN3* silencing mediated by methylation can be blocked by 5-Azacytidine (5-Aza), a DNA methyltransferase 1 (DNMT1) inhibitor, treatment in cervical cancer cell lines. Ethynyldeoxyuridine (EdU) assay, would-healing assay, clone formation assay and transwell assay were conducted to investigate the biological function of *EDN3* in cervical cancer cell lines. The results of these experiments confirmed that overexpression of *EDN3* could inhibit the proliferation, clone formation, migration and invasion of cervical cancer cells. *EDN3* may provide potential biomarker and therapeutic target for CSCC.

## Introduction

Cervical cancer (CC) is the fourth most common cancer in women worldwide (1). Approximately 604,127 new cases of cervical cancer and 341,831 related deaths in 2020 (1). More than 80% of cervical cancer related death occur in developing countries (1). In China, 47,739 deaths and 106,430 new cases of cervical cancer have been recorded, account for 18.7 percent of diagnosis and 15.3 percent of deaths globally (2). The occurrence and development of cervical cancer goes through a long pre-cancerous stage, known as cervical intraepithelial neoplasia (CIN) and graded 1-3 (3). Various risk factors that induce cervical cancer can cause early epigenetic changes and lead to abnormal gene expression, thus providing favorable conditions for the early growth of cervical cancer cells and contributing to the origin of cancer cells. Therefore, the change of epigenetics in the early stage of cervical cancer has been extensively studied (4–7).

DNA methylation is the most extensively studied epigenetic mechanism that occurs by the addition of methyl groups to specific DNA bases, typically the cytosine of CpG dinucleotides (8, 9). The addition of methyl group is catalyzed by a family of enzymes called DNA methyltransferases (DNMTs) (10, 11). DNA can be methylated throughout the genome, not only at promoter and CpG-rich regions but also at intergenic regions (12). The promoter regions of tumor suppression genes (TSG), DNA repair genes or oncogenes were frequently hypermethylated in cancers (12, 13). Abnormal methylation at promoter regions can lead to abnormal gene transcription, while abnormal levels of overall methylation are associated with many cancers (14, 15). An increasing number of studies have demonstrated the DNA methylation of specific genes, such as TSG, is associated with the pathogenesis of cervical cancer (16). Hypermethylation of specific TSG reduces the transcriptional activity of TSG, resulting in a decrease in the mRNA level of the gene’s transcriptional product and a significant decrease in the subsequent protein expression level. It indicated that the genes, down-regulated and hypermethylated in cancers, may provide potential drug targets for cervical cancer.

In order to find the novel potential genes, we analyzed all the differentially expressed genes (DEGs) in the GSE7803, GSE9750 and GSE63514 dataset. We found 67 genes were down-regulated in three datasets. Additionally, we found the expression of DNA methyltransferase 1 (DNMT1) was increased in cancer specimens in three datasets. It suggested that the change of DNA methylation must play a critical role in the development of CC. Subsequently, we used DiseaseMeth 2.0 to investigate the methylation levels of 67 down-regulated genes in normal (n=20) and cancer specimens of cervical squamous cell carcinoma (CSCC) (n=261) (17). The results showed that one of the investigated genes, endothelin 3(*EDN3*), is hypermethylated in CSCC specimens.

EDN3 is one of the endothelin (EDN) family members. There are three endothelin peptides in this family, the other two peptides are EDN1 and EDN2 (18). EDN1/2 is highly expressed in a variety of solid tumors, such as ovarian cancer, breast cancer and bladder cancer. By binding with Endothelin Receptor A (EDNRA), they can activate cell proliferation, stimulate angiogenesis, resist cell apoptosis and increase the invasion ability of cancer cells (19, 20). Unlike EDN1 and EDN2, the affinity of EDN3 and EDNRA is very low. Recent studies have demonstrated that EDN3 participate in cell proliferation, differentiation and metastasis by interacting with Endothelin Receptor B (EDNRB) (18). In breast cancer, cervical cancer, colorectal cancer and glioma, the expression of *EDN3* gene is significantly down-regulated, which may be regulated by epigenetics (21–26). *EDN3* methylation levels are high in cervical cancer patients, and *EDN3* methylation may serve as a molecular marker for cervical cancer (22, 23). However, there is no report on the role and specific mechanism of *EDN3* in cervical precancerous lesions.

In current study, we firstly detected the expression and the methylation level of *EDN3* in cervical scrapings and cervical cancer cell lines, including C-33A, SiHa and CaSki cell lines. Secondly, we the measured the expression of *EDN3* after 5-Azacytidine (5-Aza), a DNMT1 inhibitor, treatment in cervical cancer cell lines to investigate the effect of methylation on *EDN3* expression. After that, ethynyldeoxyuridine (EdU) assay, would-healing assay, clone formation assay and transwell assay were conducted to investigate the biological function of *EDN3* in cervical cancer cell lines.

## Materials and methods

### Differentially expressed genes analysis

A total of three original datasets (GSE7803, GSE9750 and GSE63514) were downloaded from Gene Expression Omnibus database (GEO; http://www.ncbi.nlm.nih.gov/geo/). GSE7803 contains 10 normal squamous cervical epitheilia samples and 21 invasive squamous cell carcinomas of the cervix samples (27). GSE9750 contains 24 normal cervix epithelium samples and 33 cervical cancers samples (28). GSE63514 contains 24 normal cervix epithelium samples, 14 CIN1, 22 CIN2, 40 CIN3 and 28 cervical cancers samples (29). Genes with adjusted *P*<0.05 and log_2_ FC < −1 (normal vs cancer) were considered as down-regulated DEGs. The overlapping DEGs were identified using jvenn online tool (http://jvenn.toulouse.inra.fr/app/index.html) (30). We found 67 genes were down-regulated in three datasets.

### Methylation analysis

The human disease methylation database (DiseaseMeth 2.0, http://www.bio-bigdata.com/diseasemeth/analyze.html) was used to investigate the methylation levels of 67 down-regulated genes in normal (n=20) and cancer specimens of CSCC (n=261) (17). This database is based on the Cancer Genome Atlas (TCGA) and GEO database. The clinical characters of patients were obtained from cBioPortal (http://www.cbioportal.org). We set the technology experimental platform as 450k (Illumina Infinium HumanMethylation 450 BeadChip), absolute methylation difference as > 0.2 and *P* value as < 0.05.

### Clinical samples

Between Jan 2021 and June 2022, cervical scrapings were collected from Xiangya Hospital of Central South University, the Second Xiangya Hospital of Central South University, the Third Xiangya Hospital of Central South University, the First Affiliated Hospital of Shantou University Medical College, Loudi Central Hospital and Zhengzhou Central Hospital Affiliated to Zhengzhou University. This study was undertaken in accordance with the Declaration of Helsinki, and the protocol was approved by the local ethical committees where applicable. All patients gave written informed consent before participation in this study. Exclusion criteria applied in this study were patients with a history of cervical cancer or existence of other cancer, cervical related-surgery, vaccinated with anti-HPV vaccine or pregnancy.

This study included women who had a normal uterine cervix (n =167), CIN1 (n=49), CIN2 (n=98), CIN3 (n=91) and CSCC (n=64) diagnosed according to the histologic reports. The final diagnosis was made by tissue-proven pathology in the CIN1+ test result group. When the biopsy results revealed CIN3+, the patients underwent cervical conization or major surgery. Methylation detection tests for *EDN3* were carried out by using residual cervical cells from cytological tests. Additionally, quantitative real-time PCR (qPCR) for *EDN3* were conducted. Only 60 normal, 29 CIN1, 37 CIN2, 57 CIN3 and 38 CSCC patients had enough residual cervical cells to isolate high-quality RNA for qPCR.

### DNA preparation

The residual cervical scrapings cells were stored in preservation solution (JIANG SU JIANYOU MEDICAL TECHNOLOGY, CHN) at −20°C. The residual cervical cells were centrifuged and washed with phosphate buffer solution (PBS). Genomic DNA (gDNA) was extracted from the cells using the PureLink Genomic DNA Mini Kit (Invitrogen, USA). The concentrations of gDNA in each sample were measured using a BioSpec-nano spectrophotometer (Shimadzu Corporation, JPN).

### Pyrosequencing

After determination of the amount of gDNA, up to 500 ng of gDNA was subjected to bisulfite conversion using ZYMO EZ DNA Methylation-Gold Kit (ZYMO RESEARCH, USA). Pyrosequencing was performed to evaluate 4 CpG sites of the *EDN3* promoter region (NC_000020.10:57875929-57875940, CGGGGCGGCGCG). PyroMark Assay Design 2.0. were used to design PCR and pyrosequencing primers. The primers were listed as follows: *EDN3*-F: 5′-GTTTGATTTAGGTTTATGGAGT-3′; *EDN3*-R: 5′- AATCCCCCCCCCTAAATCCTTTT-3′; *EDN3*-S: 5′- GTGATTTTAGTAGTAGGTAAG -3′. All the primers were produced by Shanghai Sangon Biotech Co., Ltd. Bisulfite-treated DNA was then amplified using TaKaRa Ex Taq (TaKaRa, CHN). The reaction mixture including 13.5 μl of nuclease-free water, 2 μL of 10 × Ex Taq Buffer, 2 μL of dNTP, 0.4 μL former primer and reverse primer, 0.2 μL of Ex Taq HS and 1.5 uL of bisulfite-treated DNA. The PCR product act as a template in Pyrosequencing reactions, using the PyroMark Q24 instrument (Qiagen, MD) according to the manufacturer’s recommended protocol (31). Raw data were analyzed using the Pyromark Q24 analysis software (Qiagen, MD). Pyrosequencing yields a quantitative result giving the percentage of methylated alleles for each of the 4 investigated CpG sites. The average percentage of methylation across 4 CpG sites were obtained. The average percentage of methylation of each sample was higher than 10% was regarded as methylation-positive.

### Quantitative real-time PCR

Total RNA was isolated from the tissues and cells using RNAiso Plus (TaKaRa, CHN). One microgram of total RNA was reverse-transcribed using PrimeScript™ RT reagent Kit (TaKaRa, CHN). The amplification was performed using the SYBR Green Real-Time PCR Kit (TaKaRa, CHN) on LightCycler^®^480 Instrument (Roche, CH). The primers were listed as follows: *EDN3*, F:5′-GGGACTGTGAAGAGACTGTGG-3′, R:5′-AGACACACTCCTTGTCCTTGTA-3′; β-actin, F:5′-GTGGGGCGCCCCAGGCACCA-3′, R:5′-CTCCTTAATGTCACGCACGATTTC-3′. All the primers were produced by Shanghai Sangon Biotech Co., Ltd, Shanghai, China. The qPCR was performed as described (32). Data were analyzed using the -ΔCt method and the expression of β-actin was used as normalization control.

### Cell culture and transfection

C-33A (RRID: CVCL_1094), SiHa (RRID: CVCL_0032) and CaSki (RRID: CVCL_1100) cell lines were obtained from Shanghai Institute of Biochemistry and Cell Biology of the Chinese Academy of Sciences. All cell lines were authenticated by Shanghai Biowing Applied Biotechnology Co. LTD, Shanghai, China. C-33A and SiHa cells were maintained in Minimum Essential Medium (MEM) supplemented with 10% FBS. CaSki cells was maintained in 1640 supplemented with 10% fetal bovine serum (FBS) (Gibco, USA). Cells were cultured in an incubator at 37°C and 5% CO_2_. All experiments were performed with mycoplasma-free cells. Full-length human *EDN3* cDNA was synthesized and cloned into pcDNA3.1 vector to construct the EDN3 overexpression vector (OE-*EDN3*) (Genechem, CHN). The empty pcDNA3.1 vector was used as a control. C-33A, SiHa and CaSki cell lines were seeded onto 6-well plate and transfected with OE-*EDN3* and control vector using Lipofectamine 2000 (Invitrogen, USA) according to the manufacturer’s instructions. Cells used in the following experiment were transfected with OE-*EDN3* and control vector for 48 hours.

### 5-Azacytidine treatment

After transfection, C-33A cells were seeded at 2 × 10^5^ cells/well in 6-well plate, while SiHa and CaSki cells were seeded at 1 × 10^5^ cells/well. After 24 hours later, the culture medium was replaced with fresh medium containing 5 or 10 μM 5-Aza (Selleck, CHN) or an equal volume of PBS. The cells were harvested after 24 hours.

### Western blot

Collect the cells from the cell flask into a 1.5 mL EP tube and added RIPA lysis buffer (Beyotime, CHN). The protein lysate (30 μg) was subjected to 10% SDS-PAGE and then electrotransferred onto the polyvinylidene difluoride (PVDF) membrane (Millipore IPVH00010, Solarbio, CHN). The main antibodies are as follows: β-actin (ab6276, Abcam, UK), EDN3 (H00001908-M01, Novus Biologicals, USA), the ratios of them to the primary antibody dilution buffer (Beyotime, CHN) are 1:10000 and 1:1000. The bands were washed the next day and the secondary antibodies were incubated for one hour at room temperature. The ratio of β-actin and EDN3’s secondary antibody (Proteintech, CHN) to the secondary antibody dilution buffer (Beyotime, CHN) is 1:10000 and 1:5000. The bands were washed and soaked in ECL kit (yeasen, CHN) and analyzed by ChemiDoc XRS + image analyzer (Bio-Rad, USA).

### Ethynyldeoxyuridine assay

Transfected C-33A (5000), SiHa (1000) and CaSki (1000) cells were seeded in 96-well plates. After 24 hours later, cells were pulsed with EdU and Hoechst (Cell-Light EdU Apollo567 *In Vitro* Kit, RiboBio, CHN) according to the manufacturer’s protocol. Coverslips were mounted on slides and imaged using a Thermofisher EVOS M7000 microscope. Using Image J 1.8.0 (Bethesda, USA) to quantify the positive EdU cancer cells and calculate the proliferation rate of cells.

### Wound-healing assay

Transfected cells were seeded in 6-well plate at 2 × 10^5^ cells/well (C-33A) or 1 × 10^5^ cells/well (SiHa and CaSki) for 24 hours and allowed to adhere. The cells were transfected with OE-*EDN3* and control vector as mentioned above. An acellular area was created by a 200 μL pipette tip. Photos were taken with the NIKON ECLIPSE Ts2 Microscope at 0, 48 hours and 96 hours (C-33A, SiHa), at 0, 12 hours and 24 hours (CaSki). The area of the wound was quantified by ImageJ 1.8.0 (Bethesda, USA).

### Clone formation assay

For clone formation assay, transfected C-33A cells were plated at 10000 cells per well, SiHa and CaSki cells were plated at 5000 cells per well in 6-well plates and cultured for 7 days. The complete culture medium was changed every 2 days. After 7 days, clones were fixed in 4% paraformaldehyde (Servicebio, CHN) for 30 minutes and stained with 1 mL crystal violet (Beyotime, CHN) for 1 hour. Clones containing > 50 cells were counted by Image J 1.8.0 (Bethesda, USA) for analysis.

### Transwell assay

Transwell migration assay was performed using Corning Transwell (Corning, USA). Transfected C-33A (1 × 10^6^), SiHa (3 × 10^5^) and CaSki (1 × 10^5^) cells were seeded in the upper chambers in 200 μL medium without serum. While the lower chambers were filled with 600 μL complete medium. After 48-72 hours later, the cells in the upper chambers were wiped off gently with a cotton swab. The lower cells were incubated with 600 μL 4% paraformaldehyde (Servicebio, CHN) for 30 minutes, stained with 600 μL crystal violet (Beyotime, CHN) for 1 hour and photographed under a microscope, and counted by Image J 1.8.0 (Bethesda, USA).

Transfected C-33A (2 × 10^6^), SiHa (6 × 10^5^) and CaSki (2 × 10^5^) cells were seeded in the upper chambers in 200 μL medium without serum. Invasion assay and analysis were done as mentioned above. The only difference was that the upper chambers of Corning Transwell should be coated with 80 μL ice-dissolved matrigel (Corning, USA) prior to cell invasion assay.

### Statistical analysis

All data were expressed as mean ± standard deviation (mean ± SD). Statistical analyses were performed using SPSS 24.0 or GraphPad Prism 8 (GraphPad Software, Inc., La Jolla, CA, USA). Statistical comparisons between two groups were performed using Student’s t test. The Benjamini & Hochberg (false discovery rate) method was used to adjust the *P*-value for multiple testing. All the other data were analyzed with one-way ANOVA followed by LSD (equal variances assumed) or Dunnett’s-T3 test (equal variances not assumed). χ^2^ -testing was used to analyze categorical data. All *P*-values were two-sided, and *P* < 0.05 was considered to indicate statistical significance.

## Results

### *EDN3* is downregulated and hypermethylated in CSCC

**Figure 1:**
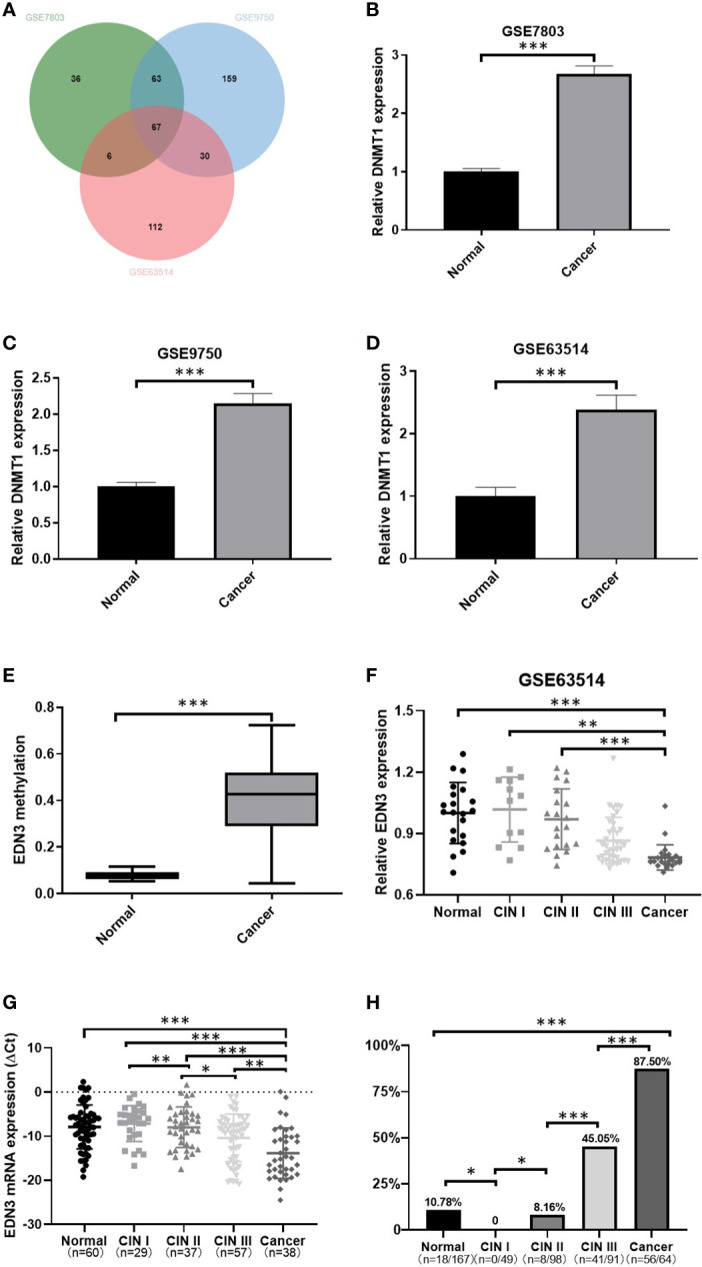
The methylation level of *EDN3* was increased and positively correlated with the severity of CIN, however, the mRNA expression level of *EDN3* was decreased and show a negative correlation with the severity of CIN. **(A)** Venn diagram of down-regulated DEGs in GSE7803, GSE9750 and GSE63514. **(B–D)** Comparison of DNMT1 expression between cancer and normal tissues. The results show that the increased expression of DNMT1 in CC tissues compared to normal tissue. **(E)** *EDN3* methylation in CSCC samples compared with that in normal samples was analyzed by the DiseaseMeth version 2.0 database. **(F)** Data from GSE63514 showed that mRNA expression of *EDN3* in CC tissues is lower than in normal and CIN1-2 tissue. **(G)** The mRNA expression of *EDN3* in our collected scrapings from patients with no CIN lesion (normal), CIN1, CIN2, CIN3 and cancer. **(H)** Bar chart showing the positive percent of *EDN3* promoter methylation levels in each histologic category. Values are mean ± SD, *P<0.05; **P<0.01; ***P<0.001.

A total of three original datasets (GSE7803, GSE9750 and GSE63514) were downloaded from GEO database. Genes with adjusted P < 0.05 and log_2_ FC < −1 (normal vs cancer) were down-regulated DEGs. Then we used an online tool, jvenn (http://jvenn.toulouse.inra.fr/app/index.html), to find overlapping down-regulated DEGs in at least two of the GEO datasets (30). We found 67 genes were down-regulated in three datasets (**Figure 1A**; **Table S1**). Additionally, we found the expression of DNMT1 was increased in cancer specimens in three datasets (**Figures 1B–D**). It suggested that the change of DNA methylation must play a critical role in the development of CC.

The genes, down-regulated and hypermethylated in cancers, may provide potential drug targets for cervical cancer. Thus, subsequently, we used DiseaseMeth 2.0 to investigate the methylation levels of 67 down-regulated genes in normal (n=20) and cancer specimens of CSCC (n=261) (17). We set the technology experimental platform as 450k (Illumina Infinium HumanMethylation 450 BeadChip), absolute methylation difference as > 0.2 and P value as < 0.05. The results showed only two investigate genes are hypermethylated in CSCC specimens, one of which, named *EDN3*, had more significant difference (**Figure 1E**).

### The expression of *EDN3* was decreased with the degree of cervical lesions, but the methylation level of *EDN3* was increased

To further investigate the expression of *EDN3* in cervical precancerous lesions, we used GSE63514 dataset and analyzed the expression of *EDN3* in different degree of cervical lesions. We found that the expression of *EDN3* was decreased with the degree of cervical lesions (**Figure 1F**). Then we measured the expression of *EDN3* in our own samples (n=221), including 60 normal, 29 CIN1, 37 CIN2, 57 CIN3 and 38 CSCC. The results are consistent with the idea that the expression of *EDN3* was decreased with the degree of cervical lesions (**Figure 1G**).

Pyrosequencing was performed to evaluate 4 CpG sites of the *EDN3* promoter region (NC_000020.10:57875929-57875940, CGGGGCGGCGCG) in our samples (n=469). The average percentage of methylation across 4 CpG sites were obtained. The average methylation percentage of each sample was higher than 10% was regarded as methylation-positive. As shown in **Figure 1H**, the methylation level of *EDN3* was increased significantly with the degree of cervical lesions, except for the normal group. The positive percent of *EDN3* promoter methylation levels in CIN1, CIN2, CIN3 and CSCC patients were 0 (0/49), 8.16% (8/98), 45.05% (41/91) and 87.50% (56/64).

### *EDN3* silencing mediated by methylation could be blocked by 5-Aza treatment in cervical cancer cell lines

**Figure 2:**
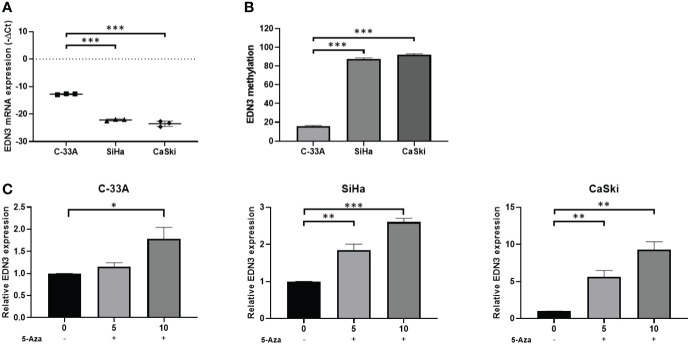
*EDN3* expression in cervical cancer cells treated with 5-Aza. **(A)** Expression levels of *EDN3* in C-33A, SiHa and CaSki cells. **(B)** Methylation levels (average percentage of methylation across 4 CpG sites) of *EDN3* in C-33A, SiHa and CaSki cells. **(C)** The mRNA expression of *EDN3* in C-33A, SiHa and CaSki cells before and after treatment with 5 and 10 μM 5-Aza. Values are mean ± SD, N = 3; *P<0.05; **P<0.01; ***P<0.001.

Firstly, we detected the expression of *EDN3* in C-33A, SiHa and CaSki cell lines. The results revealed that the expression of *EDN3* were at low level in all cervical cancer cells, especially in SiHa and CaSki cells (**Figure 2A**). Next, to investigate whether there was a correlation between *EDN3* expression and methylation in cervical cancer cell lines, we measured the methylation levels of *EDN3* by pyrosequencing. Results showed that the methylation levels of *EDN3* were at high level in all cervical cancer cells (>10%), especially in SiHa and CaSki cells (**Figure 2B**). Collectively, these data indicated that methylation might mediate silence of EDN3 in cervical cancer cell lines.

5-Aza is a DNMT1 inhibitor. The cervical cancer cell lines C-33A, SiHa and CaSki were treated with 5 or 10 μM 5-Aza or an equal volume of PBS. The expression of *EDN3* were measured after 24 hours. The results showed that the expression of *EDN3* in three cervical cancer cells was increased after 10 μM 5-Aza treatment and was only increased in SiHa and CaSki cells after 5 μM 5-Aza treatment. There was no significant difference in C-33A cells after 5 μM Aza treatment (**Figure 2C**). One explanation for this phenomenon was that the methylation level of *EDN3* was relatively low in C-33A cells. The inhibitor treatment at low concentration was not enough to cause the significant change of *EDN3* expression in C-33A. All these results indicated that there was a close correlation between EDN3 expression and methylation in cervical cancer cell lines. Furthermore, *EDN3* silencing mediated by methylation can be blocked by 5-AZA treatment in cervical cancer cell lines.

### Overexpression of *EDN3* inhibited the proliferation of C-33A, SiHa and CaSki cells.

**Figure 3:**
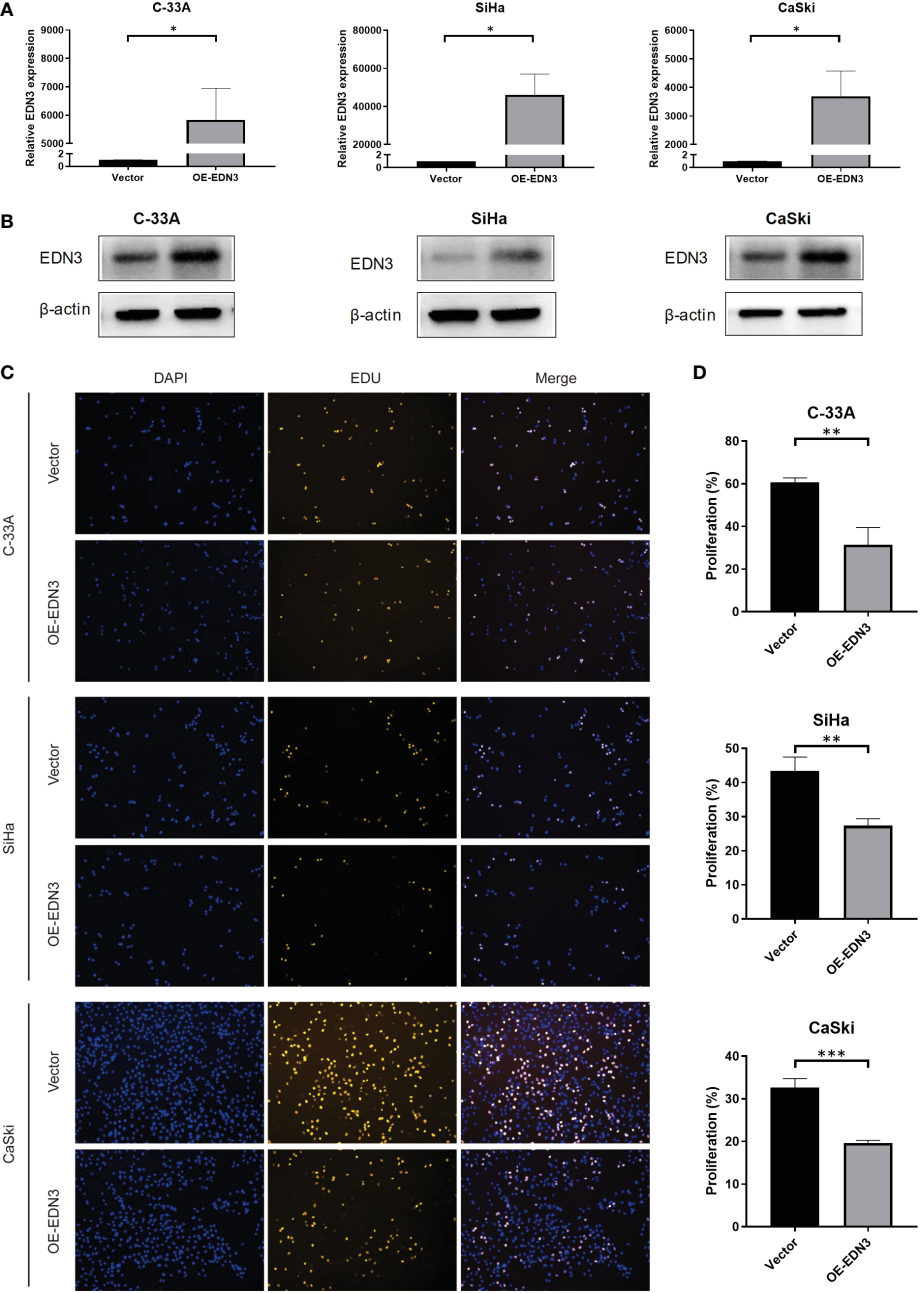
Overexpression of *EDN3* inhibited the proliferation of C-33A, SiHa and CaSki cells. **(A)** The mRNA expression of *EDN3* in C-33A, SiHa and CaSki cells transfected with OE-*EDN3* and vector. **(B)** The protein expression of *EDN3* in C-33A, SiHa and CaSki cells transfected with OE-*EDN3* and vector. **(C)** Overexpression of *EDN3* inhibited the proliferation of C-33A, SiHa and CaSki cells by EdU assay. **(D)** The proliferation percent after transfection of OE-*EDN3* and vector were measured. Values are mean ± SD, N = 3; *P<0.05; **P<0.01; ***P<0.001.

**Figure 4:**
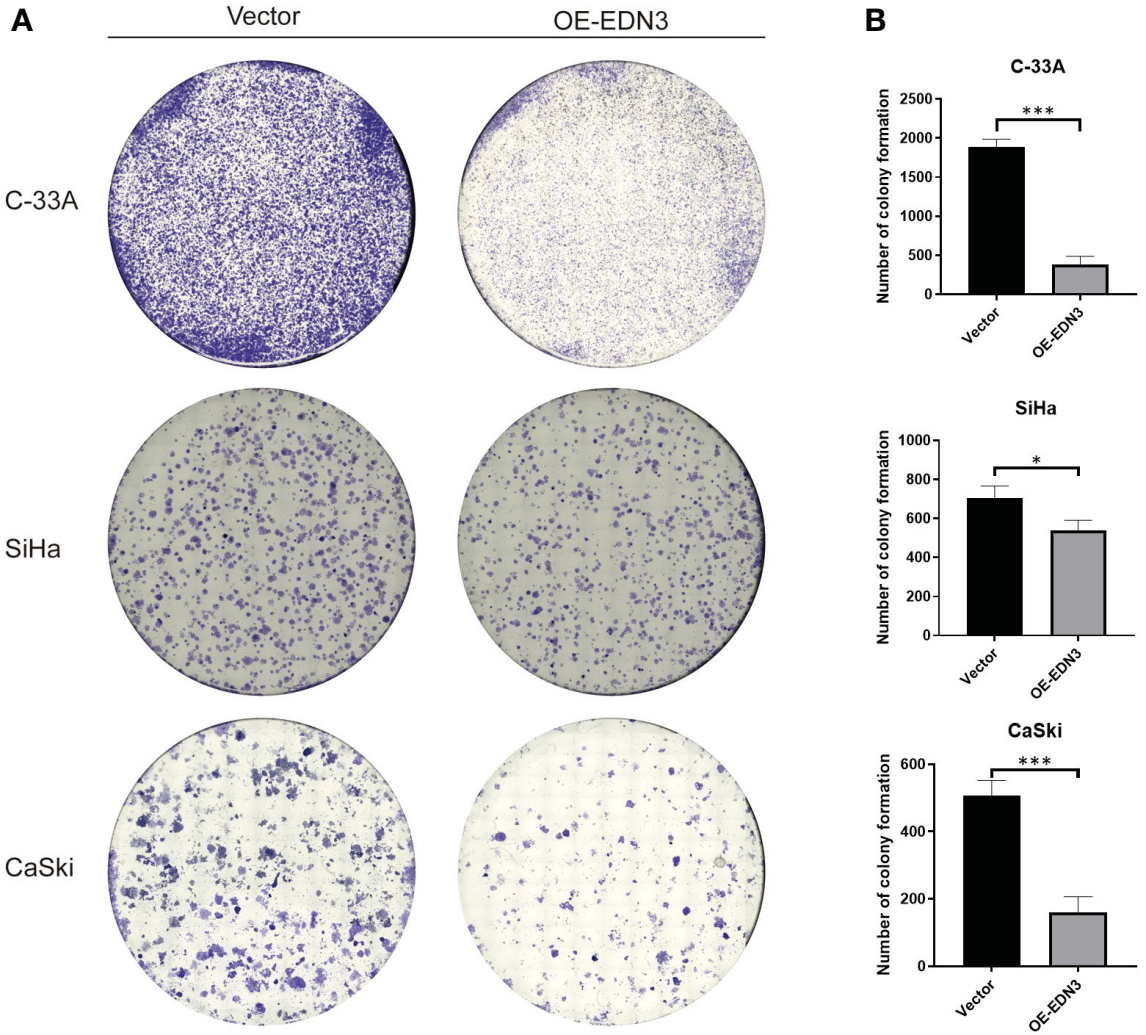
Overexpression of *EDN3* inhibited the clone formation of C-33A, SiHa and CaSki cells. **(A)** Representative images of clone formation assay using C-33A, SiHa and CaSki cells transfected with OE-*EDN3* or vector. **(B)** Quantification of clone formation assay is shown. Values are mean ± SD, N = 3; *P<0.05; ***P<0.001.

To investigate the biological functions of *EDN3* in cervical cancer cell lines, OE-*EDN3* vector was constructed. We detected the expression of *EDN3* in C-33A, SiHa and CaSki cells after transfected OE-*EDN3* or vector to verify the transfection efficiency. As the results shown in **Figures 3A, B** *EDN3* was successfully overexpressed in all cervical cancer cell lines. Subsequently, EdU and clone formation assays were conducted to assess the influence of *EDN3* on cervical cancer cell proliferation. As shown in **Figures 3C, D** and **Figure 4**, the proliferation ability and clone formation ability of cervical cancer cell lines with *EDN3* overexpression were found significantly decreased, compared with the vector, in all three cell lines.

### Overexpression of *EDN3* inhibited the migration and invasion of C-33A, SiHa and CaSki cells

**Figure 5:**
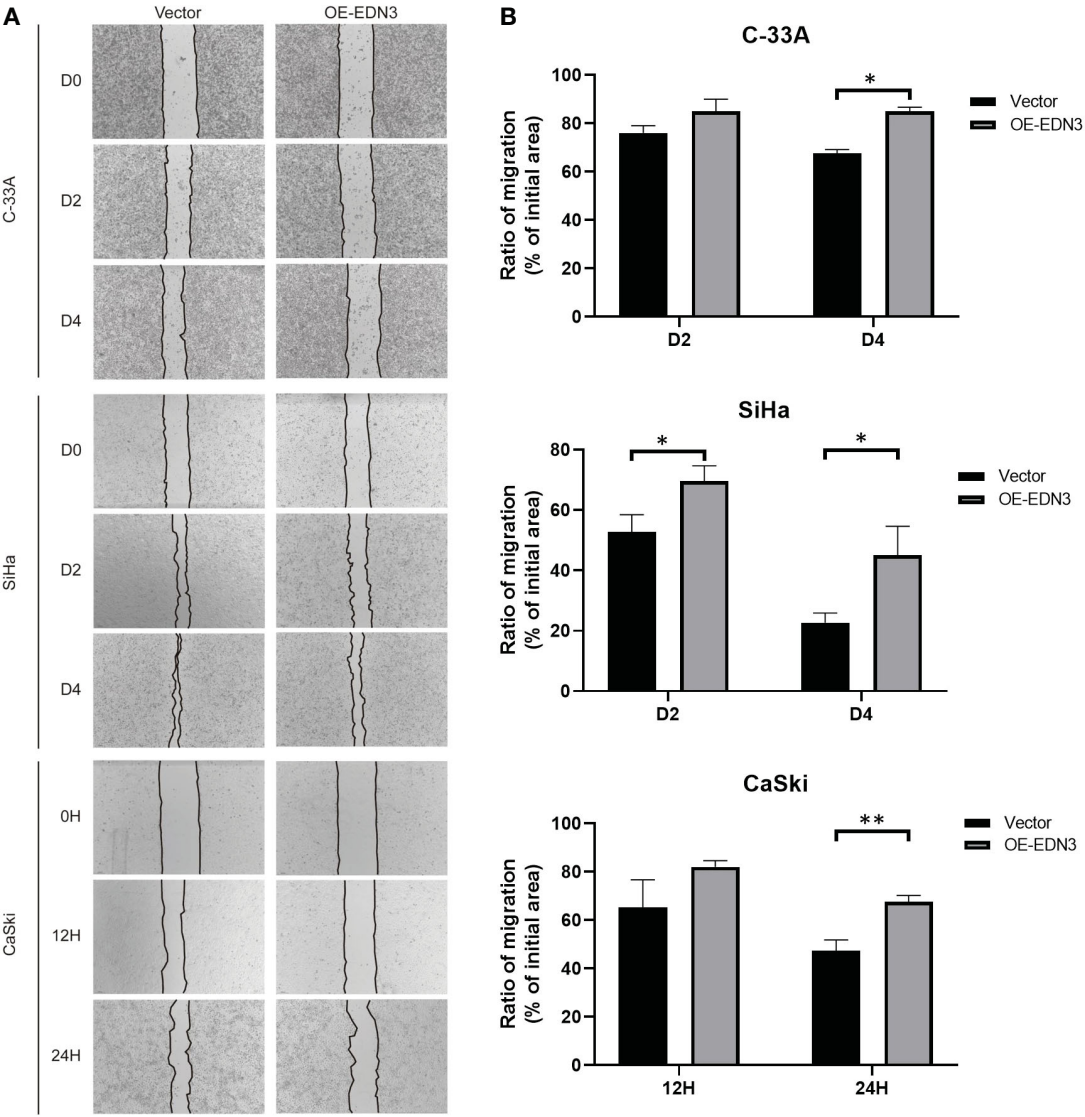
Overexpression of *EDN3* inhibited the migration of C-33A, SiHa and CaSki cells by wound healing assay. **(A)** Representative images of wound healing assay using C-33A, SiHa and CaSki cells transfected with OE-*EDN3* or vector. **(B)** Quantification of wound healing assay is shown. Values are mean ± SD, N = 3; *P<0.05; **P<0.01.

**Figure 6:**
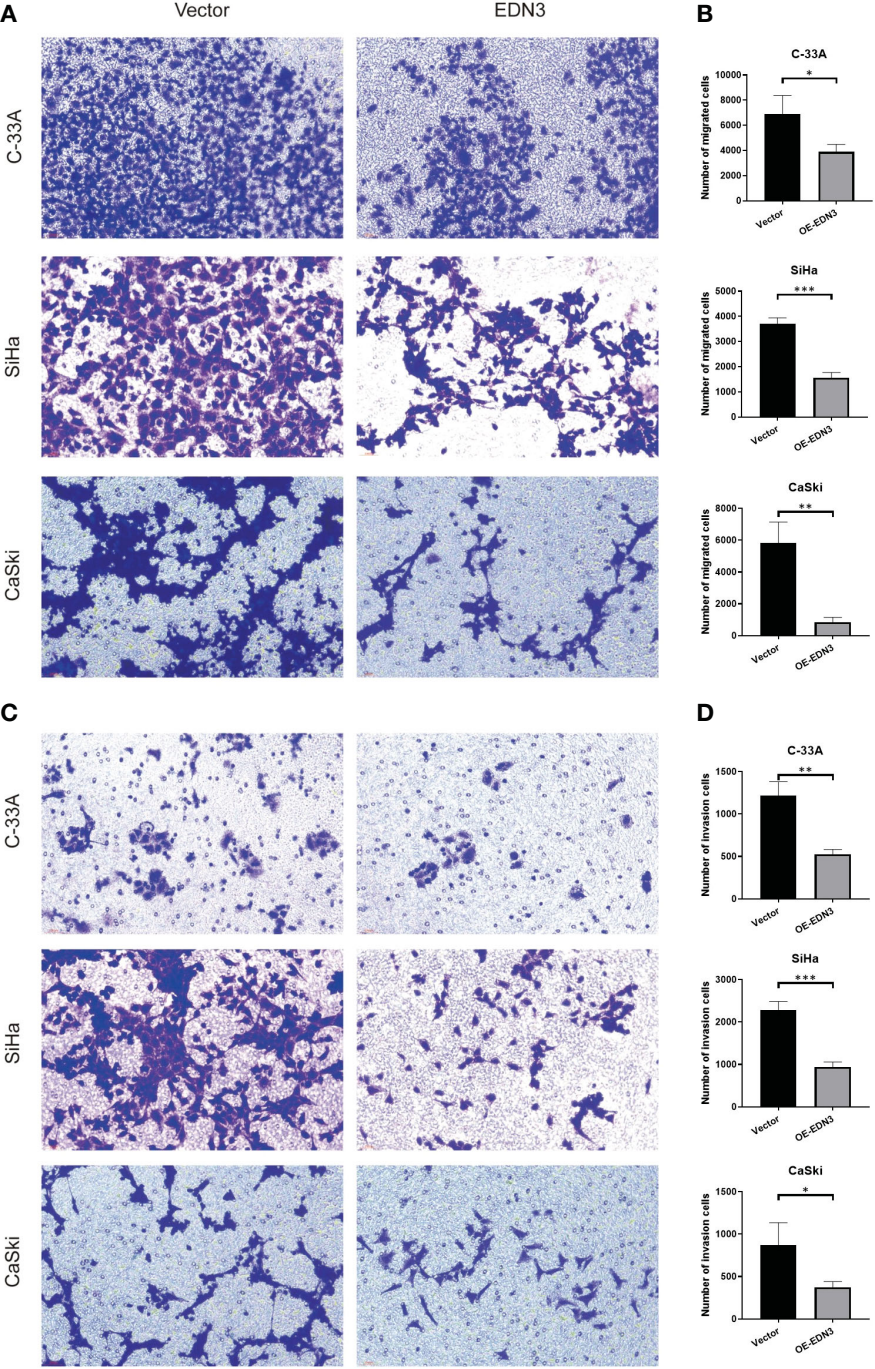
Overexpression of *EDN3* inhibited the migration and invasion of C-33A, SiHa and CaSki cells by transwell assay. **(A)** Representative images of cell migration by transwell assay using C-33A, SiHa and CaSki cells transfected with OE-EDN3 or vector. **(B)** Quantification of cell migration by transwell assay is shown. Values are mean ± SD, N = 3; *P<0.05; **P<0.01; ***P<0.001. **(C)** Representative images of cell invasion by transwell assay using C-33A, SiHa and CaSki cells transfected with OE-EDN3 or vector. **(D)** Quantification of cell invasion by transwell assay is shown. Values are mean ± SD, N = 3; *, P<0.05; **, P<0.01; ***, P<0.001.

Subsequently, we investigated that whether there is an influence of *EDN3* on cervical cancer cells migration. The wound-healing assay indicated that overexpression of *EDN3* inhibited the migration in C-33A (after 4 days), SiHa (after 2 days and 4 days) and CaSki (after 24 hours) cells (**Figure 5**). Similar results were obtained in a transwell assay (**Figures 6A, B**). In addition, the number of invasion cells were measured after 48 to 72 hours. All these results verified that the invasion capacities of cervical cancer cells in all three cell lines were significantly inhibited by overexpression of *EDN3* (**Figures 6C, D**).

## Discussion

In this study, using the datasets from GEO and TCGA databases, we found that *EDN3* was downregulated and hypermethylated in CSCC. The further analysis in GSE63514 dataset and in our own samples found that the expression of *EDN3* was decreased with the degree of cervical lesions. Pyrosequencing was performed to evaluate 4 CpG sites of the *EDN3* promoter region in our samples, the data indicated that the methylation level of *EDN3* was increased with the degree of cervical lesions. Subsequently, we found that the expression of *EDN3* was decreased, but the methylation of *EDN3* was increased in cervical cancer cell lines. *EDN3* silencing mediated by methylation can be blocked by 5-Aza, a DNMT1 inhibitor, treatment in cervical cancer cell lines. Using EdU assay, would-healing assay, clone formation assay and transwell assay, we confirmed that overexpression of *EDN3* could inhibit the proliferation, clone formation, migration and invasion of cervical cancer cells.

Increasing evidence in the past decade has demonstrated that DNA methylation in certain genes played a critical role in the progression of cervical cancer (16, 33). DNA methylation of TSGs can serve as a mechanism of carcinogenesis (34, 35). Recent studies demonstrated that many genes, such as *ADCYAP1* (36), *ASCL1* (36), *ASTN1 *(37, 38), *ATP10* (36), *CADM1 *(36), *DCC *(36), *DBC1 *(36), *DLX1 *(37, 38)*, EPB41L3 *(39), *FAM19A4* (40), *HS3ST2 *(36), *ITGA4 *(37, 38)*, JAM3 *(39), *LHX8* (41), *MAL*, *miR-124* (40), *MOS*, *MYOD1* (36), *PAX1 *(41–43), *RXFP3 *(37, 38), *SOX1*, *SOX17 *(36), *ST6GALNAC5 *(41), *TMEFF2* (36), *ZNF582* (42, 43) and ZNF671 (37, 38) et al, exhibited increased DNA methylation in cervical precancer. However, most of the research has only focused the early diagnosis function of these gene in cervical cancer screening. Researchers have paid little attention to investigate the expression change of most of these genes in different cervical lesions. Whether there is any connection between genes methylation level and expression level is unknow. Additionally, the function of these genes in cancer biology is relatively unexplored and remains largely unknown.

In our current study, detecting the expression and methylation level of *EDN3* both in clinical samples and cervical cancer cell lines, we confirmed the expression of *EDN3* was decreased, but the methylation level of *EDN3* was increased, with the degree of cervical lesions. The results in cervical cancer cell lines treated with 5-Aza further confirmed that methylation mediated silencing of *EDN3* in cervical cancer. The genes, down-regulated and hypermethylated in cancers, may provide potential drug targets for cervical cancer. We used several types of assays in this study to investigate the function of *EDN3* in cancer biology from different aspects. All results together verified that overexpression of *EDN3* could inhibit the proliferation, clone formation, migration and invasion of cervical cancer cells. It suggested that *EDN3* played a tumor suppressive function in cervical cancer. Collectively, *EDN3* is a potential biomarker and therapeutic target for CSCC. However, in this study, we only analysis the expression and methylation level of *EDN3* in CSCC samples. Whether there is any association between *EDN3* and cervical adenocarcinoma is still unknown. We should investigate that in the future research.

Recent studies reported that *EDN3* participated in cell proliferation, differentiation and metastasis in some solid tumors. In breast cancer, cervical cancer, colorectal cancer, glioma and papillary thyroid cancer, the expression of *EDN3* gene is significantly down-regulated, which may be regulated by epigenetics (21, 23–26, 44, 45). *EDN3* promotes cell apoptosis and inhibits cell invasion and migration in malignant glioma cells (21). *EDN3* is hypermethylated and down-regulated in human primary colon cancer and colon cancer cell lines, and overexpression of *EDN3* can inhibit the invasion and migration of colon cancer cells (26, 46). Abnormal methylation of *EDN3* gene is closely related to breast cancer, and hypermethylation of this gene can reduce or even silence its expression in breast cancer tissue. Patients with high expression of *EDN3* have long overall survival and disease-free survival, and *EDN3* can be used as a biomarkers for early diagnosis and prognosis of breast cancer (24, 47). In addition, reduced *EDN3* expression was associated with the progression of papillary thyroid cancer (45).

In our studies we confirmed that methylation mediated silencing of *EDN3* in cervical cancer. And this silencing mediated by methylation could be blocked by 5-Aza treatment in cervical cancer cell lines. Overexpression of *EDN3* could inhibit the proliferation, clone formation, migration and invasion of cervical cancer cells. *EDN3* played a tumor suppressive function in cervical cancer. Based on our findings, *EDN3* may serve as a potential biomarker and therapeutic target for CSCC. The detail mechanism of *EDN3* suppression of cervical cancer has not been elucidated in this study and will be further investigated in our future work.

## Data availability statement

The datasets presented in this study can be found in online repositories. The names of the repository/repositories and accession number(s) can be found in the article/**Supplementary Material**.

## Ethics statement

The studies involving human participants were reviewed and approved by the Medical Ethics Committee of Xiangya Hospital Central South University. The patients/participants provided their written informed consent to participate in this study.

## Author contributions

Designed research: QL, JX, LF, HZ, WZ, YS. Performed research: PZ, YL. Analysis data: PZ, XL. Obtained informed consent and acquired patient samples and clinical information: XL, JX, DY, LL, JH, BW, QN, SW, LD. Provided technical support: YC, SL. Wrote manuscript: PZ, XL. Revised manuscript: PZ, QL, JX. All authors contributed to the article and approved the submitted version.
