# Supplementary material for: Methylation-mediated silencing of EDN3 promotes cervical cancer proliferation, migration and invasion
Source: Front Oncol. 2023 Feb 3;13:1010132. doi: 10.3389/fonc.2023.1010132 (PMC9942821; doi:10.3389/fonc.2023.1010132)
Supplement: Supplementary file 2 [file Table_1.docx]

**Table S1 Down-regulated DEGs in GSE7803, GSE9750 and GSE63514.**

| DEG | GSE7803 | |  | GSE9750 | |  | GSE63514 | |
| --- | --- | --- | --- | --- | --- | --- | --- | --- |
|  | logFC | adj.P.Val |  | logFC | adj.P.Val |  | logFC | adj.P.Val |
| CRNN | -5.29 | 2.03E-14 |  | -6.13 | 0.00 |  | -5.51 | 1.62E-10 |
| MAL | -4.49 | 3.44E-08 |  | -6.30 | 0.00 |  | -3.95 | 1.94E-07 |
| KRT1 | -4.23 | 2.46E-10 |  | -5.82 | 0.00 |  | -2.42 | 5.16E-03 |
| UPK1A | -4.02 | 2.29E-17 |  | -4.06 | 0.00 |  | -2.56 | 2.42E-05 |
| SPINK5 | -3.85 | 2.34E-09 |  | -4.38 | 0.00 |  | -2.79 | 2.65E-06 |
| SPRR3 | -3.71 | 3.93E-06 |  | -4.32 | 0.00 |  | -2.29 | 1.04E-03 |
| DSG1 | -3.71 | 3.82E-13 |  | -4.79 | 0.00 |  | -1.93 | 3.59E-02 |
| KRT4 | -3.66 | 1.07E-07 |  | -2.52 | 0.00 |  | -1.96 | 1.75E-04 |
| CRCT1 | -3.34 | 3.76E-10 |  | -4.86 | 0.00 |  | -2.68 | 1.69E-03 |
| SOSTDC1 | -3.34 | 4.87E-18 |  | -1.90 | 0.00 |  | -2.26 | 4.12E-06 |
| ALOX12 | -3.23 | 1.23E-13 |  | -3.80 | 0.00 |  | -2.57 | 5.36E-05 |
| CRISP3 | -3.20 | 2.59E-08 |  | -5.19 | 0.00 |  | -6.49 | 8.10E-15 |
| SCEL | -2.92 | 1.49E-07 |  | -3.55 | 0.00 |  | -1.42 | 3.54E-04 |
| IVL | -2.91 | 1.95E-07 |  | -3.65 | 0.00 |  | -2.76 | 2.09E-06 |
| SPRR1A | -2.89 | 1.01E-04 |  | -3.81 | 0.00 |  | -1.99 | 4.00E-03 |
| KRT13 | -2.65 | 5.76E-04 |  | -4.08 | 0.00 |  | -3.41 | 1.14E-04 |
| ENDOU | -2.62 | 3.22E-16 |  | -3.62 | 0.00 |  | -2.72 | 1.75E-06 |
| CWH43 | -2.53 | 7.04E-18 |  | -3.51 | 0.00 |  | -2.43 | 1.24E-05 |
| SPRR1B | -2.43 | 4.73E-05 |  | -3.35 | 0.00 |  | -2.07 | 3.83E-03 |
| IL1R2 | -2.38 | 2.84E-14 |  | -1.49 | 0.00 |  | -1.89 | 1.46E-04 |
| IL18 | -2.20 | 1.59E-11 |  | -2.46 | 0.00 |  | -2.14 | 3.00E-05 |
| EMP1 | -2.17 | 1.66E-08 |  | -2.78 | 0.00 |  | -1.32 | 3.13E-05 |
| HPGD | -2.09 | 7.76E-08 |  | -2.90 | 0.00 |  | -1.90 | 2.58E-05 |
| ALOX12B | -2.03 | 1.77E-09 |  | -2.73 | 0.00 |  | -1.30 | 1.74E-02 |
| TMPRSS11E | -1.97 | 2.21E-10 |  | -3.61 | 0.00 |  | -1.32 | 3.83E-02 |
| CFD | -1.93 | 4.74E-07 |  | -1.78 | 0.00 |  | -2.10 | 3.23E-06 |
| AKR1B10 | -1.92 | 6.94E-05 |  | -2.36 | 0.00 |  | -1.93 | 9.76E-04 |
| SLURP1 | -1.88 | 1.36E-17 |  | -4.18 | 0.00 |  | -2.37 | 1.94E-07 |
| SERPINB2 | -1.88 | 3.81E-03 |  | -2.39 | 0.00 |  | -1.17 | 3.13E-02 |
| CSTB | -1.85 | 1.20E-05 |  | -1.51 | 0.00 |  | -1.20 | 1.15E-04 |
| CXCL14 | -1.82 | 9.01E-03 |  | -1.52 | 0.00 |  | -1.75 | 2.66E-05 |
| THSD4 | -1.81 | 1.54E-14 |  | -1.22 | 0.00 |  | -1.56 | 1.08E-12 |
| PPL | -1.80 | 1.25E-07 |  | -2.35 | 0.00 |  | -1.72 | 1.37E-06 |
| TGM1 | -1.77 | 3.63E-05 |  | -2.25 | 0.00 |  | -1.10 | 3.11E-02 |
| PPP1R3C | -1.77 | 3.31E-06 |  | -4.02 | 0.00 |  | -2.46 | 1.23E-06 |
| GPX3 | -1.76 | 5.89E-08 |  | -2.61 | 0.00 |  | -1.79 | 3.58E-06 |
| UPP1 | -1.76 | 2.11E-05 |  | -1.07 | 0.00 |  | -1.12 | 1.70E-03 |
| BBOX1 | -1.68 | 1.99E-10 |  | -3.16 | 0.00 |  | -2.25 | 2.57E-06 |
| LYPD3 | -1.68 | 5.74E-06 |  | -2.22 | 0.00 |  | -1.43 | 1.34E-03 |
| SERPINB1 | -1.60 | 3.29E-06 |  | -1.59 | 0.00 |  | -1.20 | 6.24E-06 |
| CYP3A7-CYP3AP1 | -1.56 | 1.77E-14 |  | -1.72 | 0.00 |  | -1.70 | 1.09E-14 |
| SLC5A1 | -1.56 | 2.66E-13 |  | -1.55 | 0.00 |  | -1.55 | 7.45E-12 |
| CLCA4 | -1.52 | 1.94E-02 |  | -3.60 | 0.00 |  | -2.83 | 5.81E-05 |
| SERPINB4 | -1.51 | 1.77E-02 |  | -1.97 | 0.01 |  | -1.90 | 1.84E-02 |
| TMPRSS11D | -1.49 | 1.76E-04 |  | -2.88 | 0.00 |  | -1.86 | 1.92E-03 |
| PTK6 | -1.47 | 2.87E-05 |  | -1.19 | 0.00 |  | -1.04 | 5.12E-04 |
| GYS2 | -1.45 | 3.84E-05 |  | -2.62 | 0.00 |  | -2.59 | 4.72E-06 |
| SERPINB3 | -1.45 | 9.98E-03 |  | -1.69 | 0.00 |  | -1.51 | 1.50E-02 |
| TP53I3 | -1.40 | 3.86E-08 |  | -1.33 | 0.00 |  | -1.65 | 3.04E-08 |
| S100A12 | -1.35 | 3.35E-04 |  | -1.34 | 0.00 |  | -1.29 | 1.16E-02 |
| **EDN3** | **-1.34** | **5.41E-13** |  | **-1.58** | **0.00** |  | **-1.65** | **4.58E-08** |
| KLK12 | -1.34 | 1.22E-12 |  | -3.18 | 0.00 |  | -1.74 | 7.36E-04 |
| ACPP | -1.30 | 2.07E-07 |  | -3.64 | 0.00 |  | -1.19 | 6.64E-05 |
| HSPB8 | -1.30 | 3.22E-05 |  | -2.35 | 0.00 |  | -1.09 | 1.81E-05 |
| PRSS3 | -1.29 | 2.85E-07 |  | -1.98 | 0.00 |  | -1.17 | 8.00E-03 |
| FMO2 | -1.25 | 3.92E-04 |  | -2.29 | 0.00 |  | -1.46 | 2.51E-04 |
| S100A9 | -1.23 | 3.83E-02 |  | -1.64 | 0.00 |  | -1.77 | 2.11E-03 |
| GPR1 | -1.18 | 1.84E-12 |  | -1.57 | 0.00 |  | -3.83 | 1.87E-29 |
| PRSS2 | -1.17 | 2.29E-12 |  | -1.46 | 0.00 |  | -1.46 | 2.40E-07 |
| ALOX15B | -1.16 | 1.33E-04 |  | -1.83 | 0.00 |  | -1.33 | 2.84E-06 |
| ZNF750 | -1.11 | 2.87E-03 |  | -2.55 | 0.00 |  | -1.69 | 9.97E-04 |
| RHCG | -1.10 | 3.13E-03 |  | -3.89 | 0.00 |  | -2.79 | 4.76E-05 |
| KLK7 | -1.07 | 8.14E-03 |  | -1.78 | 0.00 |  | -1.18 | 3.97E-03 |
| MALL | -1.05 | 4.96E-03 |  | -1.34 | 0.00 |  | -1.68 | 4.34E-05 |
| CD24 | -1.04 | 6.15E-03 |  | -1.12 | 0.00 |  | -1.05 | 2.11E-04 |
| CRYAB | -1.03 | 4.09E-06 |  | -3.21 | 0.00 |  | -1.13 | 9.75E-03 |
| SPRR2B | -1.03 | 6.61E-04 |  | -4.06 | 0.00 |  | -1.56 | 2.30E-02 |
| EREG | -3.33 | 5.34E-13 |  | -4.61 | 0.00 |  |  |  |
| HOPX | -2.80 | 1.92E-09 |  | -3.81 | 0.00 |  |  |  |
| FOSB | -1.97 | 1.71E-04 |  | -3.20 | 0.00 |  |  |  |
| KLF4 | -1.54 | 8.03E-07 |  | -3.19 | 0.00 |  |  |  |
| FCER1A | -1.96 | 5.34E-13 |  | -2.77 | 0.00 |  |  |  |
| FOS | -1.26 | 7.95E-04 |  | -2.55 | 0.00 |  |  |  |
| KRT6B | -1.47 | 8.09E-04 |  | -2.33 | 0.00 |  |  |  |
| MREG | -1.29 | 1.75E-08 |  | -2.29 | 0.00 |  |  |  |
| SPARCL1 | -2.42 | 1.48E-04 |  | -2.29 | 0.00 |  |  |  |
| GSTA4 | -1.65 | 1.67E-10 |  | -2.23 | 0.00 |  |  |  |
| SLC24A3 | -2.17 | 1.78E-11 |  | -2.23 | 0.00 |  |  |  |
| DSC2 | -1.71 | 3.68E-05 |  | -2.22 | 0.00 |  |  |  |
| GJA1 | -1.83 | 3.43E-03 |  | -2.17 | 0.00 |  |  |  |
| NOD2 | -1.31 | 2.81E-07 |  | -2.10 | 0.00 |  |  |  |
| LPAR6 | -1.22 | 8.46E-04 |  | -2.04 | 0.00 |  |  |  |
| DCN | -1.93 | 5.17E-07 |  | -1.96 | 0.00 |  |  |  |
| ZNF85 | -1.71 | 1.44E-14 |  | -1.95 | 0.00 |  |  |  |
| KRT6A | -1.10 | 2.96E-04 |  | -1.94 | 0.00 |  |  |  |
| ANXA1 | -1.58 | 1.83E-04 |  | -1.93 | 0.00 |  |  |  |
| PDGFD | -1.71 | 9.58E-10 |  | -1.91 | 0.00 |  |  |  |
| TUBB2A | -1.42 | 1.02E-03 |  | -1.90 | 0.00 |  |  |  |
| KLK6 | -1.15 | 1.56E-04 |  | -1.86 | 0.00 |  |  |  |
| GLTP | -2.09 | 5.69E-09 |  | -1.84 | 0.00 |  |  |  |
| ZNF91 | -2.00 | 3.51E-08 |  | -1.77 | 0.00 |  |  |  |
| ID4 | -1.95 | 2.98E-13 |  | -1.77 | 0.00 |  |  |  |
| RAI2 | -1.61 | 3.11E-21 |  | -1.76 | 0.00 |  |  |  |
| ABCA8 | -1.13 | 2.05E-07 |  | -1.76 | 0.00 |  |  |  |
| ZNF185 | -1.06 | 2.02E-03 |  | -1.70 | 0.00 |  |  |  |
| PELI1 | -1.28 | 1.33E-05 |  | -1.69 | 0.00 |  |  |  |
| HLA-DQB2 | -1.33 | 9.88E-05 |  | -1.66 | 0.00 |  |  |  |
| BCHE | -1.04 | 1.15E-04 |  | -1.62 | 0.00 |  |  |  |
| RND3 | -1.41 | 1.10E-03 |  | -1.60 | 0.00 |  |  |  |
| CYP3A5 | -1.97 | 2.60E-10 |  | -1.58 | 0.00 |  |  |  |
| ZFP36 | -1.71 | 1.03E-08 |  | -1.56 | 0.00 |  |  |  |
| CXCL12 | -1.39 | 7.60E-07 |  | -1.55 | 0.00 |  |  |  |
| CLDN8 | -2.11 | 1.34E-09 |  | -1.55 | 0.00 |  |  |  |
| ARMCX1 | -1.25 | 1.54E-04 |  | -1.53 | 0.00 |  |  |  |
| MAOB | -1.76 | 3.32E-19 |  | -1.49 | 0.00 |  |  |  |
| CALML5 | -1.18 | 1.04E-06 |  | -1.49 | 0.00 |  |  |  |
| SCUBE2 | -2.07 | 1.50E-18 |  | -1.47 | 0.00 |  |  |  |
| LGALSL | -1.40 | 2.00E-06 |  | -1.47 | 0.00 |  |  |  |
| TM4SF1 | -1.04 | 2.13E-03 |  | -1.43 | 0.00 |  |  |  |
| TGFBR3 | -1.46 | 3.51E-04 |  | -1.42 | 0.00 |  |  |  |
| SLC16A7 | -1.17 | 8.48E-11 |  | -1.40 | 0.00 |  |  |  |
| ZNF273 | -1.89 | 2.20E-13 |  | -1.39 | 0.00 |  |  |  |
| ISL1 | -2.70 | 1.41E-09 |  | -1.38 | 0.00 |  |  |  |
| LONRF1 | -1.09 | 2.97E-10 |  | -1.36 | 0.00 |  |  |  |
| PERP | -1.09 | 1.95E-03 |  | -1.36 | 0.00 |  |  |  |
| DEGS1 | -1.20 | 9.62E-07 |  | -1.33 | 0.00 |  |  |  |
| KLF8 | -1.09 | 1.36E-08 |  | -1.30 | 0.00 |  |  |  |
| MSMO1 | -1.40 | 2.93E-03 |  | -1.29 | 0.00 |  |  |  |
| GATM | -1.20 | 4.04E-09 |  | -1.27 | 0.00 |  |  |  |
| RHOB | -1.34 | 3.37E-03 |  | -1.23 | 0.00 |  |  |  |
| THRB | -1.13 | 9.62E-17 |  | -1.23 | 0.00 |  |  |  |
| DKK1 | -1.26 | 4.85E-03 |  | -1.22 | 0.00 |  |  |  |
| TUBA1A | -1.17 | 6.03E-04 |  | -1.22 | 0.00 |  |  |  |
| VN1R1 | -1.01 | 4.11E-16 |  | -1.18 | 0.00 |  |  |  |
| SLIT2 | -1.60 | 8.25E-15 |  | -1.17 | 0.00 |  |  |  |
| VSNL1 | -1.02 | 1.39E-03 |  | -1.17 | 0.00 |  |  |  |
| MPZL2 | -1.48 | 1.11E-07 |  | -1.12 | 0.00 |  |  |  |
| NDN | -1.71 | 1.08E-09 |  | -1.08 | 0.00 |  |  |  |
| AREG | -1.11 | 2.31E-03 |  | -1.05 | 0.00 |  |  |  |
| DCLK1 | -1.00 | 1.96E-15 |  | -1.03 | 0.00 |  |  |  |
| MGLL | -1.66 | 2.69E-11 |  |  |  |  | -1.11 | 7.75E-05 |
| BEX4 | -1.64 | 3.02E-07 |  |  |  |  | -1.14 | 1.83E-03 |
| TCN1 | -1.59 | 3.87E-03 |  |  |  |  | -1.70 | 4.99E-03 |
| SCGB2A1 | -1.32 | 3.26E-03 |  |  |  |  | -1.79 | 2.83E-02 |
| F3 | -1.16 | 3.25E-03 |  |  |  |  | -1.07 | 2.02E-03 |
| TSPO | -1.11 | 1.69E-06 |  |  |  |  | -1.27 | 7.62E-06 |
| CLIC3 |  |  |  | -3.83 | 0.00 |  | -2.24 | 2.32E-05 |
| CRABP2 |  |  |  | -2.76 | 0.00 |  | -2.07 | 5.58E-07 |
| KRT2 |  |  |  | -2.40 | 0.00 |  | -1.26 | 5.03E-03 |
| PI3 |  |  |  | -2.37 | 0.00 |  | -1.70 | 1.41E-03 |
| ZBED2 |  |  |  | -2.32 | 0.00 |  | -1.79 | 2.22E-05 |
| KLK11 |  |  |  | -2.28 | 0.00 |  | -2.43 | 2.53E-07 |
| CYP2C18 |  |  |  | -2.12 | 0.00 |  | -1.39 | 2.56E-04 |
| TGM3 |  |  |  | -2.10 | 0.00 |  | -2.16 | 5.78E-05 |
| FGFBP1 |  |  |  | -2.07 | 0.00 |  | -1.50 | 8.37E-04 |
| SLC27A6 |  |  |  | -2.04 | 0.00 |  | -1.98 | 1.43E-06 |
| CDA |  |  |  | -1.99 | 0.00 |  | -1.77 | 2.81E-06 |
| NDRG4 |  |  |  | -1.84 | 0.00 |  | -1.70 | 3.87E-05 |
| PHYHIP |  |  |  | -1.78 | 0.00 |  | -2.09 | 1.97E-16 |
| KLK10 |  |  |  | -1.76 | 0.00 |  | -1.07 | 7.88E-03 |
| NSG1 |  |  |  | -1.76 | 0.00 |  | -1.61 | 2.07E-06 |
| SULT2B1 |  |  |  | -1.73 | 0.00 |  | -2.07 | 2.45E-07 |
| KRT15 |  |  |  | -1.69 | 0.01 |  | -1.39 | 1.61E-02 |
| LOR |  |  |  | -1.60 | 0.00 |  | -1.17 | 1.48E-02 |
| S100A14 |  |  |  | -1.58 | 0.00 |  | -1.11 | 3.97E-03 |
| TRAV12-2 |  |  |  | -1.57 | 0.00 |  | -1.14 | 5.88E-16 |
| PITX1 |  |  |  | -1.52 | 0.00 |  | -1.07 | 6.07E-03 |
| FLG |  |  |  | -1.51 | 0.00 |  | -3.16 | 2.07E-05 |
| CXCR2 |  |  |  | -1.47 | 0.00 |  | -1.13 | 2.46E-03 |
| SLPI |  |  |  | -1.24 | 0.01 |  | -1.94 | 4.98E-07 |
| DEFB1 |  |  |  | -1.24 | 0.00 |  | -1.57 | 7.28E-04 |
| TRIM16 |  |  |  | -1.22 | 0.00 |  | -1.30 | 5.66E-05 |
| C1orf116 |  |  |  | -1.18 | 0.00 |  | -1.14 | 5.23E-04 |
| CCND1 |  |  |  | -1.13 | 0.00 |  | -1.54 | 3.55E-08 |
| NAP1L2 |  |  |  | -1.12 | 0.00 |  | -1.19 | 5.78E-04 |
| TST |  |  |  | -1.06 | 0.00 |  | -1.31 | 8.16E-07 |
| OLFM4 | -2.36 | 6.49E-04 |  |  |  |  |  |  |
| HLA-DQA1 | -2.00 | 1.39E-07 |  |  |  |  |  |  |
| GNG11 | -1.84 | 2.92E-17 |  |  |  |  |  |  |
| GJA5 | -1.77 | 2.90E-26 |  |  |  |  |  |  |
| COL9A1 | -1.74 | 2.28E-11 |  |  |  |  |  |  |
| LOC101927550 | -1.72 | 3.75E-23 |  |  |  |  |  |  |
| SERPINI1 | -1.70 | 1.09E-11 |  |  |  |  |  |  |
| RRAS | -1.64 | 5.37E-23 |  |  |  |  |  |  |
| FAM107A | -1.60 | 8.46E-16 |  |  |  |  |  |  |
| CNN3 | -1.57 | 2.19E-07 |  |  |  |  |  |  |
| SRPX | -1.56 | 8.13E-11 |  |  |  |  |  |  |
| DEPTOR | -1.49 | 7.10E-14 |  |  |  |  |  |  |
| TRIP12 | -1.47 | 8.14E-12 |  |  |  |  |  |  |
| PTPRZ1 | -1.40 | 2.56E-02 |  |  |  |  |  |  |
| DSCAM | -1.34 | 8.06E-17 |  |  |  |  |  |  |
| PDGFC | -1.33 | 4.62E-03 |  |  |  |  |  |  |
| SYNDIG1 | -1.33 | 7.65E-17 |  |  |  |  |  |  |
| GPR176 | -1.33 | 4.50E-20 |  |  |  |  |  |  |
| SPRY1 | -1.29 | 2.27E-10 |  |  |  |  |  |  |
| MT3 | -1.23 | 1.95E-15 |  |  |  |  |  |  |
| CRYL1 | -1.21 | 1.27E-16 |  |  |  |  |  |  |
| CACNA2D3 | -1.20 | 6.69E-04 |  |  |  |  |  |  |
| TCEAL2 | -1.20 | 1.43E-05 |  |  |  |  |  |  |
| CHST2 | -1.19 | 1.28E-11 |  |  |  |  |  |  |
| WIF1 | -1.15 | 5.98E-06 |  |  |  |  |  |  |
| BHLHE41 | -1.12 | 2.64E-05 |  |  |  |  |  |  |
| ANXA13 | -1.12 | 1.37E-20 |  |  |  |  |  |  |
| MYL3 | -1.11 | 8.00E-17 |  |  |  |  |  |  |
| LSM8 | -1.09 | 9.42E-14 |  |  |  |  |  |  |
| LDOC1 | -1.09 | 5.00E-08 |  |  |  |  |  |  |
| KIR3DS1 | -1.03 | 3.54E-14 |  |  |  |  |  |  |
| CDH22 | -1.02 | 3.53E-14 |  |  |  |  |  |  |
| FZD1 | -1.02 | 3.68E-11 |  |  |  |  |  |  |
| MSX1 | -1.01 | 6.22E-06 |  |  |  |  |  |  |
| IMPA2 | -1.01 | 3.43E-03 |  |  |  |  |  |  |
| TNFSF9 | -1.00 | 3.72E-06 |  |  |  |  |  |  |
| LY6G6C |  |  |  | -2.78 | 0.00 |  |  |  |
| SPRR2C |  |  |  | -2.52 | 0.00 |  |  |  |
| S100A7 |  |  |  | -2.49 | 0.00 |  |  |  |
| APOD |  |  |  | -2.45 | 0.00 |  |  |  |
| PTGDS |  |  |  | -2.36 | 0.00 |  |  |  |
| CSTA |  |  |  | -2.25 | 0.00 |  |  |  |
| DSG3 |  |  |  | -2.22 | 0.00 |  |  |  |
| RAB25 |  |  |  | -2.09 | 0.00 |  |  |  |
| CST6 |  |  |  | -2.07 | 0.00 |  |  |  |
| EPB41L3 |  |  |  | -1.90 | 0.00 |  |  |  |
| CLCA2 |  |  |  | -1.90 | 0.00 |  |  |  |
| KLK13 |  |  |  | -1.90 | 0.00 |  |  |  |
| KANK1 |  |  |  | -1.88 | 0.00 |  |  |  |
| TBX3 |  |  |  | -1.84 | 0.00 |  |  |  |
| BNIP3 |  |  |  | -1.83 | 0.00 |  |  |  |
| KAT2B |  |  |  | -1.82 | 0.00 |  |  |  |
| KRT10 |  |  |  | -1.82 | 0.00 |  |  |  |
| STON1 |  |  |  | -1.80 | 0.00 |  |  |  |
| KLK8 |  |  |  | -1.77 | 0.00 |  |  |  |
| DUSP1 |  |  |  | -1.75 | 0.00 |  |  |  |
| WISP2 |  |  |  | -1.75 | 0.00 |  |  |  |
| KRT14 |  |  |  | -1.74 | 0.03 |  |  |  |
| PLA2G3 |  |  |  | -1.69 | 0.00 |  |  |  |
| MEIS2 |  |  |  | -1.68 | 0.00 |  |  |  |
| PAMR1 |  |  |  | -1.67 | 0.00 |  |  |  |
| LY6D |  |  |  | -1.66 | 0.00 |  |  |  |
| ME1 |  |  |  | -1.64 | 0.00 |  |  |  |
| PLXDC2 |  |  |  | -1.63 | 0.00 |  |  |  |
| PALMD |  |  |  | -1.63 | 0.00 |  |  |  |
| TMEM45A |  |  |  | -1.61 | 0.00 |  |  |  |
| COL21A1 |  |  |  | -1.58 | 0.00 |  |  |  |
| CITED2 |  |  |  | -1.56 | 0.00 |  |  |  |
| RAB38 |  |  |  | -1.55 | 0.00 |  |  |  |
| SERPINB13 |  |  |  | -1.54 | 0.00 |  |  |  |
| ECM2 |  |  |  | -1.52 | 0.00 |  |  |  |
| TTN |  |  |  | -1.52 | 0.00 |  |  |  |
| OSR2 |  |  |  | -1.52 | 0.00 |  |  |  |
| PTGER4P2-CDK2AP2P2 | |  |  | -1.51 | 0.00 |  |  |  |
| CROT |  |  |  | -1.51 | 0.00 |  |  |  |
| PLK2 |  |  |  | -1.49 | 0.00 |  |  |  |
| BLNK |  |  |  | -1.48 | 0.00 |  |  |  |
| PDLIM2 |  |  |  | -1.48 | 0.00 |  |  |  |
| PRRX1 |  |  |  | -1.48 | 0.00 |  |  |  |
| IL1RN |  |  |  | -1.47 | 0.00 |  |  |  |
| NMU |  |  |  | -1.47 | 0.00 |  |  |  |
| TIPARP |  |  |  | -1.43 | 0.00 |  |  |  |
| SOWAHC |  |  |  | -1.42 | 0.00 |  |  |  |
| PKP1 |  |  |  | -1.41 | 0.00 |  |  |  |
| OSM |  |  |  | -1.38 | 0.00 |  |  |  |
| SASH1 |  |  |  | -1.37 | 0.00 |  |  |  |
| RANBP9 |  |  |  | -1.36 | 0.00 |  |  |  |
| TRPS1 |  |  |  | -1.36 | 0.00 |  |  |  |
| HIGD1A |  |  |  | -1.36 | 0.00 |  |  |  |
| SPINK2 |  |  |  | -1.35 | 0.00 |  |  |  |
| GHR |  |  |  | -1.35 | 0.00 |  |  |  |
| TP53AIP1 |  |  |  | -1.34 | 0.00 |  |  |  |
| DNASE1L3 |  |  |  | -1.34 | 0.00 |  |  |  |
| GEM |  |  |  | -1.34 | 0.00 |  |  |  |
| CD207 |  |  |  | -1.34 | 0.00 |  |  |  |
| CALML3 |  |  |  | -1.32 | 0.00 |  |  |  |
| TPD52L1 |  |  |  | -1.31 | 0.00 |  |  |  |
| SMPD2 |  |  |  | -1.31 | 0.00 |  |  |  |
| EGR2 |  |  |  | -1.31 | 0.00 |  |  |  |
| ZNF43 |  |  |  | -1.30 | 0.00 |  |  |  |
| PKP3 |  |  |  | -1.30 | 0.00 |  |  |  |
| DPT |  |  |  | -1.29 | 0.00 |  |  |  |
| DKK2 |  |  |  | -1.29 | 0.00 |  |  |  |
| HMGCS1 |  |  |  | -1.28 | 0.00 |  |  |  |
| ABCA12 |  |  |  | -1.27 | 0.00 |  |  |  |
| MAFF |  |  |  | -1.26 | 0.00 |  |  |  |
| SMPDL3A |  |  |  | -1.26 | 0.00 |  |  |  |
| SMAGP |  |  |  | -1.26 | 0.00 |  |  |  |
| AHNAK2 |  |  |  | -1.26 | 0.00 |  |  |  |
| SPRY2 |  |  |  | -1.26 | 0.00 |  |  |  |
| SEPP1 |  |  |  | -1.26 | 0.00 |  |  |  |
| FSCN3 |  |  |  | -1.25 | 0.00 |  |  |  |
| CD36 |  |  |  | -1.25 | 0.00 |  |  |  |
| DSP |  |  |  | -1.25 | 0.00 |  |  |  |
| EGR3 |  |  |  | -1.25 | 0.00 |  |  |  |
| CEACAM7 |  |  |  | -1.25 | 0.00 |  |  |  |
| CMA1 |  |  |  | -1.24 | 0.00 |  |  |  |
| HBEGF |  |  |  | -1.24 | 0.00 |  |  |  |
| OVOL1 |  |  |  | -1.24 | 0.00 |  |  |  |
| PLBD1 |  |  |  | -1.24 | 0.00 |  |  |  |
| TGM5 |  |  |  | -1.20 | 0.00 |  |  |  |
| SFRP4 |  |  |  | -1.20 | 0.01 |  |  |  |
| ATP10B |  |  |  | -1.19 | 0.00 |  |  |  |
| ATP10D |  |  |  | -1.19 | 0.00 |  |  |  |
| PLD1 |  |  |  | -1.18 | 0.00 |  |  |  |
| ECM1 |  |  |  | -1.18 | 0.00 |  |  |  |
| PDZD2 |  |  |  | -1.17 | 0.00 |  |  |  |
| ABI3BP |  |  |  | -1.17 | 0.00 |  |  |  |
| PTPRN |  |  |  | -1.17 | 0.00 |  |  |  |
| HMGCR |  |  |  | -1.16 | 0.00 |  |  |  |
| EGR1 |  |  |  | -1.16 | 0.00 |  |  |  |
| YOD1 |  |  |  | -1.15 | 0.00 |  |  |  |
| CLTB |  |  |  | -1.15 | 0.00 |  |  |  |
| COL17A1 |  |  |  | -1.15 | 0.00 |  |  |  |
| METTL7A |  |  |  | -1.14 | 0.00 |  |  |  |
| NPY1R |  |  |  | -1.14 | 0.00 |  |  |  |
| AR |  |  |  | -1.14 | 0.00 |  |  |  |
| F10 |  |  |  | -1.14 | 0.00 |  |  |  |
| TRIM29 |  |  |  | -1.14 | 0.00 |  |  |  |
| AQP1 |  |  |  | -1.14 | 0.00 |  |  |  |
| ADRB2 |  |  |  | -1.14 | 0.00 |  |  |  |
| CLCN3 |  |  |  | -1.13 | 0.00 |  |  |  |
| CCNG2 |  |  |  | -1.13 | 0.00 |  |  |  |
| SPAG16 |  |  |  | -1.13 | 0.00 |  |  |  |
| DOK3 |  |  |  | -1.13 | 0.00 |  |  |  |
| JAM2 |  |  |  | -1.13 | 0.00 |  |  |  |
| TOB1 |  |  |  | -1.13 | 0.00 |  |  |  |
| ADAM18 |  |  |  | -1.12 | 0.00 |  |  |  |
| SFRP1 |  |  |  | -1.12 | 0.00 |  |  |  |
| MYC |  |  |  | -1.11 | 0.00 |  |  |  |
| DHRS1 |  |  |  | -1.11 | 0.00 |  |  |  |
| AQP3 |  |  |  | -1.11 | 0.00 |  |  |  |
| KLF6 |  |  |  | -1.10 | 0.00 |  |  |  |
| TRHDE |  |  |  | -1.10 | 0.00 |  |  |  |
| MLLT4 |  |  |  | -1.10 | 0.00 |  |  |  |
| SLC39A2 |  |  |  | -1.10 | 0.00 |  |  |  |
| C12orf29 |  |  |  | -1.10 | 0.00 |  |  |  |
| A1CF |  |  |  | -1.10 | 0.00 |  |  |  |
| DBI |  |  |  | -1.09 | 0.00 |  |  |  |
| DPP6 |  |  |  | -1.08 | 0.00 |  |  |  |
| MAP2K4 |  |  |  | -1.08 | 0.00 |  |  |  |
| ITM2A |  |  |  | -1.07 | 0.00 |  |  |  |
| FABP5 |  |  |  | -1.07 | 0.00 |  |  |  |
| SLC19A2 |  |  |  | -1.07 | 0.00 |  |  |  |
| ZSCAN18 |  |  |  | -1.06 | 0.00 |  |  |  |
| RAPGEFL1 |  |  |  | -1.06 | 0.00 |  |  |  |
| IGF1 |  |  |  | -1.06 | 0.00 |  |  |  |
| UBL3 |  |  |  | -1.05 | 0.00 |  |  |  |
| SLCO2A1 |  |  |  | -1.05 | 0.00 |  |  |  |
| MXD1 |  |  |  | -1.05 | 0.00 |  |  |  |
| RAB11A |  |  |  | -1.05 | 0.00 |  |  |  |
| VAMP8 |  |  |  | -1.04 | 0.00 |  |  |  |
| WISP3 |  |  |  | -1.04 | 0.00 |  |  |  |
| LAMB2 |  |  |  | -1.04 | 0.00 |  |  |  |
| MAST4 |  |  |  | -1.04 | 0.00 |  |  |  |
| OGN |  |  |  | -1.03 | 0.01 |  |  |  |
| ZSCAN31 |  |  |  | -1.03 | 0.00 |  |  |  |
| ISOC1 |  |  |  | -1.03 | 0.00 |  |  |  |
| EPHX3 |  |  |  | -1.03 | 0.00 |  |  |  |
| KCNJ15 |  |  |  | -1.03 | 0.00 |  |  |  |
| HEBP2 |  |  |  | -1.03 | 0.00 |  |  |  |
| ARG1 |  |  |  | -1.03 | 0.01 |  |  |  |
| CDHR1 |  |  |  | -1.03 | 0.00 |  |  |  |
| PPFIBP2 |  |  |  | -1.03 | 0.00 |  |  |  |
| DPP4 |  |  |  | -1.03 | 0.00 |  |  |  |
| PIM1 |  |  |  | -1.02 | 0.00 |  |  |  |
| GUCY1A2 |  |  |  | -1.02 | 0.00 |  |  |  |
| DAAM1 |  |  |  | -1.02 | 0.00 |  |  |  |
| ACAT1 |  |  |  | -1.02 | 0.00 |  |  |  |
| SGK1 |  |  |  | -1.01 | 0.00 |  |  |  |
| MAFB |  |  |  | -1.01 | 0.00 |  |  |  |
| ZNF107 |  |  |  | -1.01 | 0.00 |  |  |  |
| SGMS1 |  |  |  | -1.00 | 0.00 |  |  |  |
| PEG3 |  |  |  | -1.00 | 0.00 |  |  |  |
| CBR4 |  |  |  | -1.00 | 0.00 |  |  |  |
| TMPRSS11B |  |  |  |  |  |  | -4.16 | 5.86E-07 |
| CRISP2 |  |  |  |  |  |  | -3.97 | 8.53E-16 |
| SPINK7 |  |  |  |  |  |  | -3.52 | 1.74E-05 |
| SBSN |  |  |  |  |  |  | -3.16 | 3.82E-05 |
| KRTDAP |  |  |  |  |  |  | -3.02 | 1.13E-03 |
| PNLIPRP3 |  |  |  |  |  |  | -2.68 | 6.76E-06 |
| GJB2 |  |  |  |  |  |  | -2.65 | 1.11E-05 |
| CNFN |  |  |  |  |  |  | -2.64 | 2.04E-04 |
| C1orf177 |  |  |  |  |  |  | -2.56 | 1.52E-09 |
| BPIFB1 |  |  |  |  |  |  | -2.51 | 1.93E-04 |
| SFTA2 |  |  |  |  |  |  | -2.48 | 1.50E-05 |
| CYSRT1 |  |  |  |  |  |  | -2.47 | 1.19E-06 |
| DMKN |  |  |  |  |  |  | -2.46 | 7.82E-06 |
| LCN2 |  |  |  |  |  |  | -2.44 | 1.24E-05 |
| GJB6 |  |  |  |  |  |  | -2.41 | 1.14E-03 |
| RPTN |  |  |  |  |  |  | -2.37 | 2.38E-04 |
| KRT78 |  |  |  |  |  |  | -2.37 | 7.41E-07 |
| FAM3D |  |  |  |  |  |  | -2.34 | 3.44E-11 |
| LCE3D |  |  |  |  |  |  | -2.33 | 1.32E-03 |
| PSCA |  |  |  |  |  |  | -2.30 | 2.44E-07 |
| DAPL1 |  |  |  |  |  |  | -2.28 | 1.47E-04 |
| FCGBP |  |  |  |  |  |  | -2.11 | 1.94E-05 |
| TFF3 |  |  |  |  |  |  | -2.09 | 9.24E-04 |
| PRAC1 |  |  |  |  |  |  | -2.08 | 3.00E-06 |
| TPRG1 |  |  |  |  |  |  | -2.07 | 2.10E-04 |
| RBM20 |  |  |  |  |  |  | -2.06 | 1.16E-09 |
| LOC100130476 |  |  |  |  |  |  | -2.05 | 1.48E-04 |
| LOC100507221 |  |  |  |  |  |  | -1.95 | 7.11E-08 |
| RDH12 |  |  |  |  |  |  | -1.94 | 1.79E-04 |
| GCNT3 |  |  |  |  |  |  | -1.90 | 2.28E-05 |
| PRSS27 |  |  |  |  |  |  | -1.83 | 1.61E-04 |
| SDR9C7 |  |  |  |  |  |  | -1.80 | 5.26E-06 |
| ZG16B |  |  |  |  |  |  | -1.80 | 2.86E-06 |
| LOC102724689 |  |  |  |  |  |  | -1.77 | 5.58E-05 |
| LOC101927313 |  |  |  |  |  |  | -1.76 | 1.40E-22 |
| SCGB2A2 |  |  |  |  |  |  | -1.72 | 4.30E-03 |
| PCP4L1 |  |  |  |  |  |  | -1.67 | 1.65E-04 |
| MUC5B |  |  |  |  |  |  | -1.65 | 1.06E-05 |
| SULT1E1 |  |  |  |  |  |  | -1.64 | 6.61E-05 |
| C10orf99 |  |  |  |  |  |  | -1.60 | 4.25E-03 |
| MAB21L3 |  |  |  |  |  |  | -1.59 | 1.40E-03 |
| LOC102659288 |  |  |  |  |  |  | -1.59 | 3.74E-03 |
| CYP4F22 |  |  |  |  |  |  | -1.56 | 1.25E-06 |
| SCNN1B |  |  |  |  |  |  | -1.54 | 5.31E-06 |
| SCGB1D2 |  |  |  |  |  |  | -1.54 | 6.94E-03 |
| A2ML1 |  |  |  |  |  |  | -1.52 | 4.27E-04 |
| C5orf66-AS1 |  |  |  |  |  |  | -1.51 | 7.99E-04 |
| C19orf33 |  |  |  |  |  |  | -1.49 | 2.36E-04 |
| GBP6 |  |  |  |  |  |  | -1.47 | 5.63E-03 |
| PROM1 |  |  |  |  |  |  | -1.46 | 2.01E-02 |
| ZNF334 |  |  |  |  |  |  | -1.45 | 6.19E-12 |
| VSIG10L |  |  |  |  |  |  | -1.42 | 1.10E-02 |
| MUC15 |  |  |  |  |  |  | -1.41 | 4.21E-03 |
| TMEM79 |  |  |  |  |  |  | -1.41 | 4.51E-05 |
| PIGR |  |  |  |  |  |  | -1.41 | 3.40E-04 |
| HS3ST6 |  |  |  |  |  |  | -1.41 | 1.62E-04 |
| SLC16A9 |  |  |  |  |  |  | -1.41 | 3.53E-06 |
| KRT32 |  |  |  |  |  |  | -1.38 | 5.42E-15 |
| MYZAP |  |  |  |  |  |  | -1.38 | 1.35E-04 |
| ZNF439 |  |  |  |  |  |  | -1.37 | 1.77E-07 |
| PP14571 |  |  |  |  |  |  | -1.34 | 2.19E-08 |
| CYP3A43 |  |  |  |  |  |  | -1.34 | 7.92E-15 |
| SPINK6 |  |  |  |  |  |  | -1.30 | 8.81E-03 |
| HP |  |  |  |  |  |  | -1.29 | 5.20E-03 |
| CYP4X1 |  |  |  |  |  |  | -1.29 | 1.18E-02 |
| SPATA16 |  |  |  |  |  |  | -1.27 | 1.82E-21 |
| PDZK1IP1 |  |  |  |  |  |  | -1.26 | 4.65E-03 |
| DMBT1 |  |  |  |  |  |  | -1.24 | 7.67E-05 |
| SH3PXD2A-AS1 | |  |  |  |  |  | -1.24 | 3.60E-06 |
| PLLP |  |  |  |  |  |  | -1.24 | 1.71E-05 |
| RAET1E |  |  |  |  |  |  | -1.24 | 2.55E-06 |
| ZNF426 |  |  |  |  |  |  | -1.24 | 2.28E-05 |
| PCDHB2 |  |  |  |  |  |  | -1.23 | 1.65E-03 |
| TMEM40 |  |  |  |  |  |  | -1.23 | 3.57E-05 |
| MUC5AC |  |  |  |  |  |  | -1.23 | 1.36E-05 |
| ATP6V1C2 |  |  |  |  |  |  | -1.19 | 5.34E-03 |
| C15orf52 |  |  |  |  |  |  | -1.18 | 1.33E-04 |
| ST6GALNAC1 |  |  |  |  |  |  | -1.18 | 1.56E-02 |
| AKR1C3 |  |  |  |  |  |  | -1.18 | 3.70E-02 |
| WFDC5 |  |  |  |  |  |  | -1.17 | 1.02E-03 |
| NRTN |  |  |  |  |  |  | -1.17 | 2.32E-03 |
| C15orf48 |  |  |  |  |  |  | -1.17 | 2.99E-02 |
| SOX17 |  |  |  |  |  |  | -1.16 | 4.60E-02 |
| ZNF662 |  |  |  |  |  |  | -1.16 | 6.13E-05 |
| FZD10 |  |  |  |  |  |  | -1.15 | 1.15E-03 |
| LINC00284 |  |  |  |  |  |  | -1.13 | 1.11E-03 |
| FUT3 |  |  |  |  |  |  | -1.13 | 7.22E-07 |
| TFF1 |  |  |  |  |  |  | -1.11 | 4.00E-04 |
| KRT23 |  |  |  |  |  |  | -1.11 | 3.19E-02 |
| SNORA68 |  |  |  |  |  |  | -1.10 | 1.82E-09 |
| FOLR1 |  |  |  |  |  |  | -1.10 | 5.77E-03 |
| PLA2G4A |  |  |  |  |  |  | -1.09 | 2.60E-02 |
| TMEM132C |  |  |  |  |  |  | -1.09 | 3.93E-09 |
| ZNF404 |  |  |  |  |  |  | -1.08 | 3.77E-04 |
| ZNF667-AS1 |  |  |  |  |  |  | -1.08 | 1.95E-03 |
| CYP2B7P |  |  |  |  |  |  | -1.08 | 4.27E-04 |
| MPST |  |  |  |  |  |  | -1.07 | 1.21E-07 |
| SERPINB11 |  |  |  |  |  |  | -1.07 | 7.14E-04 |
| ACOX2 |  |  |  |  |  |  | -1.05 | 3.51E-06 |
| CDH13 |  |  |  |  |  |  | -1.05 | 2.43E-04 |
| SYTL1 |  |  |  |  |  |  | -1.04 | 3.02E-05 |
| SCNN1G |  |  |  |  |  |  | -1.04 | 1.58E-06 |
| CLDN10 |  |  |  |  |  |  | -1.03 | 2.13E-02 |
| TMEM45B |  |  |  |  |  |  | -1.03 | 8.20E-03 |
| RHOD |  |  |  |  |  |  | -1.03 | 1.50E-05 |
| GALNT5 |  |  |  |  |  |  | -1.02 | 1.63E-04 |
| MIR31HG |  |  |  |  |  |  | -1.02 | 4.47E-05 |
| SHISA6 |  |  |  |  |  |  | -1.02 | 4.86E-03 |
| DLX3 |  |  |  |  |  |  | -1.02 | 3.64E-07 |
| BTBD11 |  |  |  |  |  |  | -1.00 | 5.30E-04 |
| CREG2 |  |  |  |  |  |  | -1.00 | 2.00E-05 |
| ANKRD35 |  |  |  |  |  |  | -1.00 | 2.33E-03 |
